# Assessing the Usability of a Prescription-Based Mobile App for Patients With Panic Disorder and Its Management Console for Clinicians: Controlled User Study

**DOI:** 10.2196/76843

**Published:** 2025-10-17

**Authors:** Yujin Ko, Jeemin Lee, Kyunghee Ham, Yesol Cho, Yu-Bin Shin, Choongki Min, Kyungnam Kim, Wonseuk Jang, Hayeon Jung, Jae-Jin Kim

**Affiliations:** 1 Institute of Behavioral Sciences in Medicine, Yonsei University College of Medicine, Seoul, Republic of Korea; 2 Department of Psychiatry, Soonchunhyang University Bucheon Hospital, Bucheon, Republic of Korea; 3 AI Medtech R&D, Waycen Inc., Seoul, Republic of Korea; 4 Yonsei University Gangnam Severance Hospital Medical Device Usability Research Center, Seoul, Republic of Korea; 5 Department of Psychiatry, Yonsei University Gangnam Severance Hospital, 211 Eonju-ro, Gangnam-gu, Seoul, 06273, Republic of Korea, 82 01090259219

**Keywords:** panic disorder, digital therapeutics, mobile application, usability testing, cognitive behavioral therapy, patient-centered design, clinician-guided care, real-time intervention, lifestyle modification

## Abstract

**Background:**

Panic disorder is characterized by sudden panic attacks and persistent anticipatory anxiety. While pharmacotherapy remains effective, patients with panic disorder often experience residual symptoms and functional impairments. Lifestyle factors influence symptom severity but are often unaddressed in routine psychiatric care. Most current digital therapeutics for panic disorder have a limited scope, lack integration with clinicians, and fail to consider behavioral patterns. To address these limitations, our research team developed a prescription-based app that supports structured cognitive behavioral therapy practice, real-life symptom management, and lifestyle modifications for patients with panic disorder, and a management console—a web-based platform that allows clinicians to monitor the patients’ engagement and progress as well as determine therapeutic options if necessary.

**Objective:**

This study aimed to test the usability of the app and management console by evaluating their interface, functionality, and user experience. The primary goal was to identify the strengths and areas for further improvement of these software devices and to develop a list of modifications to improve the user experience and clinical applicability in updates to refine the devices for a future clinical trial.

**Methods:**

Usability data were collected by investigators at a medical device usability research center without the involvement of the development research team, and the participants were 15 patients with panic disorder and 15 psychiatrists. Each group completed experimental use of the app or management console and scored the convenience and safety of its modules, questionnaire evaluations for the acceptability, and presentation of verbal subjective feedback on areas for improvement. Based on the participants’ suggestions, a list of items that need to be modified to improve functionality and ease of use for each device was created.

**Results:**

Patients completed 155 assigned tasks for the app with more than 98% success, and psychiatrists completed 34 tasks for the management console with more than 86% success. The convenience and safety scores for the app and management console exceeded the neutral threshold (mean >4.5). For all statements about the acceptability, both patients and psychiatrists responded at the level of agreeing with a score exceeding 3 (mean: 3.6~4.3 and 4~4.7, respectively). There were 38 suggestions for app improvements and 66 suggestions for management console improvements, most of which were incorporated in the modification list.

**Conclusions:**

Patients reported that the app might be easy to use and help manage anxiety, and psychiatrists found the management console practical and well-suited for outpatients. By combining patient-facing therapeutic tools with clinician-driven prescription and monitoring, the devices offer a solution aligned with clinically integrated, real-world psychiatric care. Modified devices based on the improvement suggestions presented in this study will be evaluated in future clinical trials for their impact on engagement and treatment outcomes.

## Introduction

Panic disorder is one of the most common disorders worldwide, characterized by unexpected and recurrent panic attacks, along with intense fear, arousal, and a sense of losing control [1]. These symptoms can significantly lower the quality of life, disturb daily functioning, and even result in avoidance behaviors. Panic disorder has a lifetime prevalence of approximately 1% to 4%, making it a large public health burden [2].

While pharmacotherapy remains the primary treatment modality, cognitive behavioral therapy (CBT) has also proven efficacy [3,4]. CBT is a structured psychotherapy that helps patients identify and modify maladaptive thought processes and behavior patterns. For panic disorder, CBT involves psychoeducation, cognitive restructuring, interoceptive and situational exposure, and breathing retraining [5]. However, in real-world settings, patients often continue to experience frequent panic attacks in their daily lives with limited access to timely or structured psychological support beyond medication. Real-world practice of CBT remains limited by the requirement of frequent visits, unavailability of trained therapists, and practical challenges such as patients’ time constraints or geographic limitations [6]. In addition, lifestyle factors such as caffeine intake, alcohol consumption, physical activity, and sleep patterns have been shown to influence the course of panic disorder symptoms [7-10]. Despite their importance, these factors are often left unaddressed in standard care, leaving patients to manage them on their own without proper guidance.

In recent years, digital therapeutics (DTx) have emerged as a novel approach for providing psychological interventions in a more accessible and convenient format. Recent studies have shown the effectiveness of DTx for panic disorder. For example, Freespira, approved by the US Food and Drug Administration (FDA) for the treatment of panic disorder, has been shown to be effective in reducing symptoms by training patients to regulate their respiration rate and CO_2_ exhalation, with improvements lasting for up to 12 months after treatment [11]. PanicMechanic (Amshuhu iTech Solution Pvt. Ltd.), a smartphone–based app, provides real-time biofeedback during actual panic attacks by monitoring heart rate through the camera sensor, offering support in daily life situations [12]. In another recent study, a mobile CBT-based app designed for panic disorder showed improvement of symptoms in more than 80% of participants, using a combination of psychoeducation, journaling, various tasks, and gamified content [13].

Despite the advancement of DTx for panic disorder, most still fall short of providing a comprehensive approach that includes cognitive, behavioral, physiological, and lifestyle-related factors [14]. A common limitation of clinician-facing instruments is the limited availability that allows physicians to prescribe treatment, monitor progress, and offer feedback [15]. For these platforms to be adopted into standard psychiatric care, especially within time-limited outpatient practice, their functionality should allow for real-time monitoring of symptoms and meaningful patient-clinician communication. These demands highlight the need for an enhanced and integrated DTx model that unites structured CBT, an immediate panic attack support system, a lifestyle management system, and a dedicated clinician interface.

To address this need, we developed Waymed_panic (Waycen Inc.), a system consisting of a prescription-based DTx app and web-based platform to treat panic disorder. This study tested the usability of the app and the management console by evaluating their interface, functionality, and users’ experience among patients with panic disorder and psychiatrists. The primary goal of this usability test was to identify the strengths and areas for further improvement of these software devices. Through this test, we aimed to develop a list of modifications to improve the users’ experience and clinical applicability in updates to refine the devices for a future clinical trial.

## Methods

### Overview

**Figure 1.**
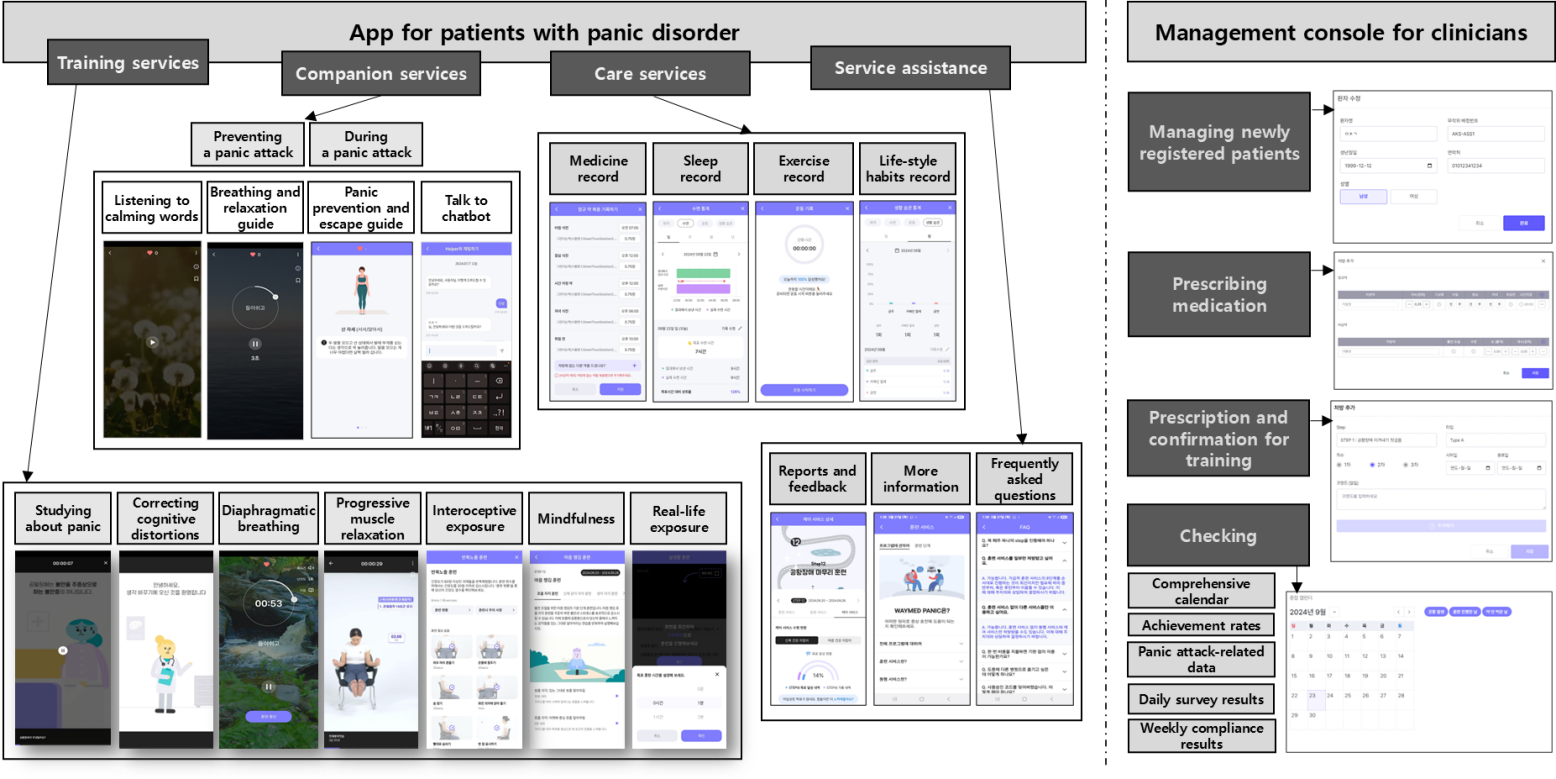
Illustration showing the overall structure of Waymed_panic. The app consists of 3 major services—training, companion, and care—and a service assistance section. It also includes a management console through which clinicians can prescribe the app, review patient records, and provide feedback.

This study was performed to evaluate participant perspectives of usability and satisfaction with Waymed_panic, which consists of a therapeutic app that provides training, companionship, and care services for patients with panic disorder and a management console for clinicians (Figure 1). A detailed description of their contents is provided in Multimedia Appendix 1. The primary goal of the app is to improve acute panic symptoms through the delivery of CBT training, real-time coping support, and lifestyle modification and management tools, all within a user-friendly mobile interface. In parallel, the web-based platform allows clinicians to monitor the patients’ engagement and progress and to determine therapeutic options, if necessary. This system aims to bridge the gap between stand-alone mobile interventions and integrated psychiatric care by combining self-guided digital tools with clinician-directed oversight.

This system was developed in approximately 2 years as a collaborative project by a multidisciplinary research team. The medical content was developed by psychiatrists working at university hospitals based on clinical evidence and treatment guidelines for panic disorder [16-18], and all content components followed evidence-based principles. Clinical psychologists researching at a medical school research center assisted in the initial design and creation of the CBT content and provided specialist consultation on clinical materials and composition. While developing the therapeutic content, major clinical guidelines and key literature on CBT for panic disorder [5,19] were referenced, and core services were structured based on established intervention models [6,20-22]. User interface and user experience designers from a health care app development company developed a user-friendly, intuitive interface that was easy to navigate and visually clear. App programmers from the same company built the technical structure for the app, developed both Android and iOS versions, and integrated features. The team focused on refining the prototype and adjusting the user flow to ensure clinical and functional consistency through regular meetings for determining the design of a stepwise unlocking approach, the structure of panic-time interventions, and a web-based console for clinicians. In this study, participant recruitment and evaluation were conducted by investigators at a medical device usability research center, without involvement of the development research team.

### Participants

Patients with panic disorder were recruited through advertisements posted in the psychiatric outpatient clinic of the Yonsei University Gangnam Severance Hospital, and psychiatrists were recruited through an advertisement posted on the internet-based community of psychiatrists affiliated with the Yonsei University Health System. Inclusion criteria for the patient group were adults aged 19 to 59 years, diagnosed with panic disorder according to *DSM-V* (*The Diagnostic and Statistical Manual of Mental Disorders, Fifth Edition*) criteria through a medical history interview with a psychiatrist, and able to use mobile apps manually, hear speech, read and write Korean, understand visual elements such as signs and symbols, and detect type, size, shape, and color of visual stimuli. Inclusion criteria for psychiatrists were the same as for patients, except that they had to be a psychiatrist who treats panic disorder instead of being diagnosed with panic disorder. Through those advertisements, 21 patients and 16 psychiatrists were recruited, and among them, 15 patients (7 males and 8 females; mean age 32.9, SD 10.9 years) and 15 psychiatrists (10 males and 5 females; mean age 33.3, SD 5.4 years) were the final study participants, who met the above criteria and agreed to participate in the study.

### Procedure

All participants visited the laboratory of the Yonsei University Gangnam Severance Hospital Medical Device Usability Research Center, where they completed experimental use of the software device, questionnaire evaluations, and presentation of verbal subjective opinions on areas for improvement. They first watched a preprepared educational video that introduced patients to the app and psychiatrists to the management console.

Then, they had at least 30 minutes to actually try out the app installed on a smartphone (Samsung Galaxy S20) or the management console installed on a tablet computer (Samsung Galaxy Tab A8). During these experimental uses, they performed the tasks executing the contents of the app and management console at the request of the investigator. Each execution task was presented with a visual projection and an oral reading from the investigator in parallel to ensure that the participants could easily understand the corresponding instruction. A different number of execution tasks was given to the patient participants for each of the modules that make up the app (ie, running the app, training services, companion services, care services, and service assistance functions), and the total number of tasks was 155 (Multimedia Appendix 2). Execution tasks were not applied to all modules to ensure effective progress of the investigation. For example, since among the training services, progressive muscle relaxation has similar aspects to other trainings and real-life exposure is not suitable for conducting in a laboratory setting, these 2 trainings were not included as execution tasks to prevent the overall task execution time from becoming too long. Execution tasks were also given to the psychiatrist participants in different numbers for each of the modules that make up the management console (ie, running the console, managing newly registered patients, prescribing medication, prescription and confirmation for training, and checking the comprehensive calendar, achievement rates, panic attack–related data, daily survey results, and weekly compliance results), and the total number of the tasks was 34 (Multimedia Appendix 3).

The investigator observed the participants’ behavior related to performing each execution task and recorded whether the task was completed or not. In principle, participants performed the tasks on their own without any assistance from the investigator, but if they requested help due to difficulty performing a task or failed to complete it for more than 60 seconds, the investigator provided assistance. Both of these instances were considered task failures and were reflected in the calculation of the task success rate. As each execution task was completed, the participants responded to a 5-point Likert scale to rate the convenience (1: very difficult, 2: somewhat difficult, 3: neutral, 4: somewhat easy, and 5: very easy) and safety (1: very dangerous, 2: slightly dangerous, 3: neutral, 4: slightly safe, and 5: very safe) of the task.

After completing all the execution tasks, the participants responded to a questionnaire to assess the acceptability of the app or management console, which consisted of 10 different statements (Table 1) about its use on a 5-point Likert scale (1: strongly disagree, 2: somewhat disagree, 3: neutral, 4: somewhat agree, and 5: strongly agree). These statements were prepared by modifying Nielsen’s usability heuristics [23] to suit the contents of each device, and additional references in these modifications were the System Usability Scale [24] for the app and the Post-Study System Usability Questionnaire [25] for the management console. In addition, the investigator recorded the participants’ verbal subjective opinions about areas for improvement due to the difficulties they encountered in each module while performing the execution tasks and also recorded the overall verbal opinions on areas for improvement and satisfaction with the app or management console.

**Table 1.** Statements to evaluate acceptability of the app and management console, and the acceptability scores in patients with panic disorder and psychiatrists.

Statements	Score^a^, mean (SD)
For patients (n=15)	
The system is easy to use.	4.1 (0.6)
The system does not require any technical assistance to use.	4.1 (0.9)
The system is not unnecessarily complicated.	3.7 (1)
The system provides useful information about the training outcomes.	4.3 (0.7)
The system offers a wealth of options for a variety of features.	4.3 (0.8)
The system is designed with easy-to-navigate content and functional organization.	3.9 (0.9)
The system is designed to fit into an environment in which CBT^b^ is practiced.	4.1 (0.8)
The system is designed to fit into a situation in which a panic attack may occur.	3.6 (1)
The system is designed to be aesthetically pleasing.	3.8 (1)
The system is designed to have a proper layout.	3.6 (0.9)
For psychiatrists (n=15)	
The system is designed with a list page to make it easy to find a patient of interest.	4.5 (0.7)
This system has an appropriate response speed when a button is clicked.	4.2 (0.9)
This system provides an appropriate font size for the patient details page.	4.4 (0.9)
This system is designed to facilitate understanding of the patient’s training outcomes.	4.4 (0.8)
The system is designed to facilitate the prescribing of services to patients.	4.6 (0.9)
The system is designed with easy-to-navigate layout of the service history.	4 (1.1)
The system makes it easy to understand patient’s lifestyle information at a glance.	4.2 (0.7)
This system makes it easy to understand the information the graph represents.	4.6 (0.6)
The system is designed to be aesthetically pleasing.	4.7 (0.6)
The system is satisfactory in terms of overall user experience.	4.5 (0.6)

a Responses were scored on a 5-point Likert scale (1: strongly disagree, 2: somewhat disagree, 3: neutral, 4: somewhat agree, and 5: strongly agree). Higher numbers indicate greater agreement with the statement.

b CBT: cognitive behavioral therapy.

### Analysis

An index of whether participants successfully performed a given execution task was the task success rate, which was calculated for each module based on the investigator’s observation data and represents the percentage of participants who completed the task relative to the total number of participants. The convenience and safety scores for each module were calculated as the mean (SD) of the Likert scale scores for the subjective data of the participants. In addition, to determine which content received many suggestions for improvement, the percentage of participants who provided suggestions relative to the total number of participants was calculated for each module and referred to as the feedback rate. The results of the questionnaire about the acceptability of the app and management console were referred to as the acceptability score, which was calculated as the mean (SD) of Likert scale scores for each statement. The scores of convenience, safety, and acceptability were checked to see if they exceeded the set target of 3 or higher for each execution task or each acceptability statement.

### Modifications After Usability Testing

In response to the suggestions for improvement from the participants, a list of items that need to be modified to improve functionality and ease of use in the structure and progress of the app or management console was created. The suggestion reflection rate (SRR) was calculated as an indicator of how many of these suggestions were actually reflected in this modification list, which was the percentage of suggestions that were actually incorporated in the modification list among the total suggestions.

### Ethical Considerations

The study protocol was approved by the Yonsei University Gangnam Severance Hospital Institutional Review Board (3-2024-0117). Signed written informed consent was obtained from all participants after a detailed explanation of the study procedure and data privacy and confidentiality by an investigator with good clinical practice training. All data collected and used for analysis were anonymized, and therefore, this study does not contain any personally identifiable images or information. The participation fee was KRW 250,000 (approximately US $210) for patients and KRW 300,000 (approximately US $250) for psychiatrists, which were paid via bank transfer within 1 month of participating in the study.

## Results

### Execution of the App

Table 2 presents the results of 155 app execution tasks in total for 15 patients with panic disorder, divided by module. For all modules, the patients with panic disorder successfully performed the executive tasks at a high rate of more than 98%. In terms of the convenience score, which assessed how easy it was to execute, they responded that all individual modules were easy to execute, with a score exceeding 3 (an average score of 4.7 to 5). In terms of the safety score, which evaluated how safe it was to execute, they also responded that individual modules were safe to execute, with a score exceeding 3 (an average score ranging from 4.7 to 4.9). Among the modules, “Diaphragmatic breathing” of the training service, “During a panic attack” of the companion service, and “Sleep record” of the care service received many suggestions for improvement with a feedback rate of more than 25%, whereas “Correcting cognitive distortions” and “Interoceptive exposure” of the training service, and “More information” and “Frequently asked questions” of the service assistance received no suggestion for improvement with a feedback rate of 0%.

**Table 2.** Task success rates, convenience scores, and safety scores for module-specific execution tasks in the usability evaluation of the app by patients with panic disorder (n=15).

Modules	Number of execution tasks	Task success rate (%)	Convenience score^a^, mean (SD)	Safety score^b^, mean (SD)	Feedback rate (%)
Running the app	3	100	4.8 (0.4)	4.7 (0.5)	6.7
Training service					
Studying about panic	9	100	4.9 (0.3)	4.9 (0.4)	6.7
Correcting cognitive distortions	14	100	4.9 (0.4)	4.8 (0.5)	0
Diaphragmatic breathing	27	100	4.8 (0.6)	4.8 (0.4)	46.7
Interoceptive exposure	11	99.4	4.8 (0.5)	4.8 (0.4)	0
Mindfulness	21	99.7	4.7 (0.8)	4.8 (0.6)	20
Companion service					
Preventing a panic attack	5	100	4.7 (0.6)	4.8 (0.5)	20
During a panic attack	16	100	4.8 (0.6)	4.8 (0.5)	33.3
Care service					
Medicine record	10	98	4.7 (0.7)	4.7 (0.6)	6.7
Sleep record	8	99.1	4.7 (0.6)	4.7 (0.6)	26.7
Exercise record	11	98.2	4.9 (0.4)	4.8 (0.6)	20
Service assistance					
Reports and feedback	4	100	4.7 (0.7)	4.8 (0.6)	6.7
More information	3	100	4.9 (0.3)	4.9 (0.3)	0
Frequently asked questions	3	100	5 (0.2)	4.9 (0.3)	0

a Responses were scored on a 5-point Likert scale (1: very difficult, 2: somewhat difficult, 3: neutral, 4: somewhat easy, and 5: very easy).

b Responses were scored on a 5-point Likert scale (1: very dangerous, 2: slightly dangerous, 3: neutral, 4: slightly safe, and 5: very safe).

### Execution of the Management Console

Table 3 shows the results of a total of 34 console execution tasks for 15 psychiatrists, divided by module. For all modules, the psychiatrists successfully performed the executive tasks at a high rate of more than 86%. In terms of the convenience score, they responded that all individual modules were easy to execute with a score exceeding 3 (an average score of 4.3 to 4.9). In terms of the safety score, they also responded that all individual modules were safe to execute with a score exceeding 3 (an average score ranging from 4.7 to 4.9). Among the modules, “Managing newly registered patients,” “Prescribing medication,” “Prescription and confirmation for training,” “Checking: Comprehensive calendar,” and “Checking: Achievement rates” received many suggestions for improvement with a feedback rate of more than 25%, whereas “Running the console” and “Checking: Weekly compliance results” received no suggestion for improvement with a feedback rate of 0%.

**Table 3.** Task success rates, convenience scores, and safety scores for module-specific execution tasks in the usability evaluation of the management console by psychiatrists (n=15).

Modules		Number of execution tasks	Task success rate (%)	Convenience score^a^, mean (SD)	Safety score^b^, mean (SD)	Feedback rate (%)
Running the console		2	100	4.8 (0.5)	4.8 (0.6)	0
Managing newly registered patients		4	95	4.6 (0.8)	4.7 (0.6)	60
Prescribing medication		5	98.6	4.8 (0.6)	4.7 (0.7)	46.7
Prescription and confirmation for training		9	97.8	4.7 (0.7)	4.8 (0.4)	33.3
Checking						
Comprehensive calendar		2	96.5	4.3 (1.1)	4.7 (0.6)	40
Achievement rates		6	86.7	4.3 (1)	4.8 (0.4)	53.3
Panic attack–related data		2	87	4.8 (0.4)	4.9 (0.3)	20
Daily survey results		2	100	4.8 (0.5)	4.9 (0.3)	6.7
Weekly compliance results		2	100	4.9 (0.3)	4.9 (0.3)	0

a Responses were scored on a 5-point Likert scale (1*: *very difficult, 2: somewhat difficult, 3: neutral, 4: somewhat easy, and 5: very easy).

b Responses were scored on a 5-point Likert scale (1: very dangerous, 2: slightly dangerous, 3: neutral, 4: slightly safe, and 5: very safe).

### Acceptability

Table 1 shows the acceptability scores for our system surveyed by the participants. For all 10 different statements that the app was appropriately designed, the patients with panic disorder responded at the level of “somewhat agreeing” with a score exceeding 3. That is, for 5 items, the acceptability scores were slightly higher than 4, and for 5 items, they were slightly lower than 4. For all 10 different statements about the management console being appropriately designed, the psychiatrists also gave the acceptability scores above 3. They were 4.4 or lower, near the level of “somewhat agree” for 5 items, and 4.5 or higher, near the level of “strongly agree” for the remaining 5 items.

### Modification List for Upgrading the Functionality

Table 4 presents the number of suggestions for improvement, SRR for system modifications, and examples of the modifications by each module of the app and management console. There were a total of 38 suggestions from the 14 patient participants, almost all of which were reflected in the modification list for upgrading the app functionality, resulting in a 100% SRR in 7 modules. However, 3 suggestions, 1 each for “Mindfulness” of the training service, “Preventing a panic attack” of the companion service, and “Medication record” of the care service, were not reflected for legal or technical reasons, and thus the SRR for these modules did not reach 100%. Meanwhile, all 15 psychiatrist participants made at least 1 suggestion, for a total of 66 suggestions, all of which were reflected in the modification list to upgrade the console functionality, resulting in a 100% SRR across all modules.

**Table 4.** Number of improvement suggestions, suggestion reflection rate, and example of system modifications by each module of the app and management console.

Modules	Number	SRR^a^ (%)	Examples of modification
App			
Running the app	1	100	Change the member ID from an email to a mobile phone number.
Training service			
Studying about panic	1	100	Add a [NEXT] icon to move on to the next step after answering a quiz.
Correcting cognitive distortions	0	Not applicable	Not applicable
Diaphragmatic breathing	8	100	Improve readability in the explanation of the importance and ease of breathing.
Interoceptive exposure	0	Not applicable	Not applicable
Mindfulness	4	75	Change the options items to be displayed on 1 screen without scrolling.
Companion service			
Preventing a panic attack	7	86	Improve the quality of the screen images in breathing from low to high quality.
During a panic attack	7	100	Change the system so that the written panic symptom information can be used for the input.
Care service			
Medicine record	1	0	Not applicable
Sleep record	7	100	Add an icon to allow the wake-up time to be entered automatically.
Exercise record	1	100	Change the actual exercise time input screen to be more visible through auto-scrolling.
Service assistance			
Reports and feedback	1	100	Improve the readability of the reports in the section that tracks training progress.
More information	0	Not applicable	Not applicable
Frequently asked questions	0	Not applicable	Not applicable
Management console			
Running the console	0	Not applicable	Not applicable
Managing newly registered patients	13	100	Change the method of entering the patient’s date of birth from using the calendar to entering it directly.
Prescribing medication	10	100	Add a status table to show medication history at a glance.
Prescription and confirmation for training	12	100	Modify moving between months to be possible via scrolling.
Checking			
Comprehensive calendar	12	100	Revise the colors in the comprehensive calendar to indicate what they mean.
Achievement rates	14	100	Modify the status of goal achievement in ”Care Service” to be checked by week.
Panic attack–related data	4	100	Modify the statistics to be viewed on a daily basis rather than a weekly basis.
Daily survey results	1	100	Modify the positioning of scores on the anxiety and depression graphs to become more visible.
Weekly compliance results	0	Not applicable	Not applicable

a SRR: suggestion reflection rate.

## Discussion

### Principal Findings

In this study, we carried out formative usability testing in patients with panic disorder and psychiatrists to evaluate the practicality, applicability, and embeddedness of the therapeutic app and management console in a clinical setting. Both groups demonstrated high scores in all measures, including task success rate, convenience score, safety score, and acceptability score, confirming the satisfactory usability of the devices. They also offered meaningful qualitative feedback on product improvements, which allowed us to develop a list of modifications to improve users’ experience and clinical applicability, and to implement updates to further enhance the devices.

For the therapeutic app, patients successfully completed a total of 155 execution tasks across all modules with a success rate of more than 98%, and each module received an average score of greater than 3 on both convenience (≥4.7) and safety (≥4.7), suggesting that the app was easy and safe to execute and navigate. Regarding statements assessing the acceptability of the app, patients also responded that it was appropriately designed, with a level of “somewhat agree.” Despite these high scores, the app received the lowest score in terms of relevance to panic attack situations and layout appropriateness. Because panic attack situations vary greatly from patient to patient, it is difficult to fully reflect this diversity in the app, and thus, improvements in this area will likely be limited. However, the layout appropriateness is expected to improve significantly if patient feedback is actively incorporated. Overall, patients appear to perceive the app as effectively designed and easy to use. We believe these findings are consistent with previous notions that navigation clarity, modular content structure, and access to real-time support features are important factors determining patient engagement and satisfaction levels [26].

Although patients demonstrated these high task success rates and positive responses, they still identified areas in need of improvement, providing a total of 38 suggestions. Except for a few suggestions that could not be implemented due to legal or technical issues, we incorporated most of them into our modification list. When categorizing these suggestions by module, 4 modules, such as “Diaphragmatic Breathing” in the training service, “Preventing a panic attack” and “During a panic attack” in the companion service, and “Sleep Recording” in the care service, received a relatively high number of suggestions (7 or 8) from patients, which were related to weaknesses in visual guidance and complex multistep interactions. These issues were considered important in developing the modification list, as they could increase cognitive load, especially when used in acutely anxious situations. In contrast, patients gave no suggestions for 2 modules, such as “Correcting Cognitive Distortions” and “Interoceptive Exposure” in the training service, suggesting that cognitively structured and self-paced components in these modules were more straightforward to engage with.

Previous studies have demonstrated that patients who receive a combination of CBT, behavioral interventions, and lifestyle modification achieve better treatment outcomes than those who receive a single modality approach [27,28]. Moreover, patients prefer apps that provide immediate assistance in moments of distress [28] and also require guidance in everyday life, such as reducing caffeine intake or sustaining regular physical activity, which traditional care often fails to provide [29]. Therefore, once the issues summarized in the modification list are addressed, our mobile DTx app, which can provide a variety of treatment modules, may be considered to be a novel treatment option for panic disorder.

For the management console, psychiatrist participants successfully completed a total of 34 execution tasks across all modules with a success rate of more than 86%, and each module received an average score of greater than 3 on both convenience (≥4.3) and safety (≥4.7), suggesting that the management console was easy and safe to execute. Psychiatrists also rated the console’s acceptability highly, giving it a mid-range assessment between “somewhat agree” and “strongly agree.” Taken together, they seem to perceive the management console as well-designed and easy to use.

Although psychiatrist participants gave high task success rates and positive evaluation on the management console, they all provided 66 suggestions for usability improvements, which was much more than those from patient participants. Most of these improvement suggestions focused on modules related to new patient management and service prescribing, reflecting clinicians’ priorities for establishing control when using digital platforms. They were particularly interested in features that enable them to efficiently manage patients and prescribe interventions flexibly based on monitoring progress in a clinically meaningful manner.

Since these improvement suggestions did not raise any legal or technical issues for implementation, we included all of them in the modification list, and the production of a management console with improved functionality will follow. We believe that these modifications will help the management console better align with the routine psychiatric care flow. While previous DTx usability testing focused on the patient perspective [30,31], this study incorporated psychiatrists assessing the usability of the management console. Unlike commercial mental health apps that users download without any barriers to access and manage themselves, our system is initiated by a clinician’s prescription within the context of ongoing psychiatric care. The requirement for a prescription makes the functionality of the management console crucial, which is why it was included in the current usability testing. This prescription-based model provides not only a medical framework but also therapeutic legitimacy, encouraging patients to perceive the program as a formal part of their treatment, much like taking a prescription medication. The fact that app usage can be monitored by the prescribing clinician can increase patient trust and compliance. Clinicians can also review a patient’s app usage profiles to develop a follow-up treatment plan, thereby enhancing the effectiveness and justification of subsequent prescriptions.

### Limitations

This study was only the first step in determining how well patients with panic disorder and their clinicians could use our DTx system. This study was conducted at a single site with a small sample size, limiting its generalizability. While participants were representative of the intended user base, the testing environment may not fully capture the complexity of real-world use. Because our system encompasses a significant number of services and functions, such as how many modules will be used by patients in a real-world setting and how engagingly the system will be used between clinicians and patients in clinical settings, could be crucial for usability. However, since this study is insufficient to draw any conclusions on these issues, further real-world research is needed to address them.

### Conclusions

The results of this study suggest that Waymed_panic, consisting of a therapeutic app and management console, is sufficiently easy and acceptable to use for the treatment of panic disorder. A distinction of this system from many commercially available apps is the integration of therapeutic content with clinician engagement, enabling not only structured CBT practices, but also real-time coping support, lifestyle tracking, and reciprocating feedback between the clinician and patient. These contents highlight the growing demand for digital resources that integrate treatment into everyday life. With ever-increasing demands and burdens on the mental health system, using integrated DTx models that can extend capacity without compromising quality or personalization might be a way to address the national shortfall of accessible mental health care. To be used in this way, randomized clinical trials are first needed to determine the practical efficacy and safety of this system in treating various symptoms of panic disorder.

## Supplementary material

10.2196/76843 Multimedia Appendix 1 Core features and functions of the app and management console.

10.2196/76843 Multimedia Appendix 2 Execution tasks of the app for patients with panic disorder.

10.2196/76843 Multimedia Appendix 3 Execution tasks of the management console for psychiatrists.

## Abbreviations

CBT: cognitive behavioral therapy
*DSM-V*: *The Diagnostic and Statistical Manual of Mental Disorders, Fifth Edition*
DTx: digital therapeutics
FDA: US Food and Drug Administration
SRR: suggestion reflection rate
