# Supplementary material for: Assessing the Usability of a Prescription-Based Mobile App for Patients With Panic Disorder and Its Management Console for Clinicians: Controlled User Study
Source: JMIR Form Res. 2025 Oct 17;9:e76843. doi: 10.2196/76843 (PMC12533929; doi:10.2196/76843)
Supplement: Multimedia Appendix 1 [file formative-v9-e76843-s001.docx]

**[Appendix 1] Core features and functions of the app and management console**

**1. The app for patients with panic disorder**

***Training services***

The Training services deliver a progressive 8-step CBT-based program composed of seven modules. Each module is structured based on treatment goals, with content such as video lectures, interactive quizzes (e.g., OX format), or self-monitoring worksheets. Patients retain access to completed modules for review. The modules include:

- Module A. Studying about Panic: Psychoeducation that provides foundational information about panic disorder, its symptoms, mechanisms, and treatment rationale. This module has four sub-modules to be completed over a four-week period, including watching three videos on understanding panic disorder and panic attacks (A1), two videos on the causes and symptoms of panic disorder (A2), three videos on medications for treating panic disorder (A3), and two videos on the course and prognosis of panic disorder (A4).
- Module B. Correcting Cognitive Distortions: Cognitive restructuring that helps patients recognize and modify maladaptive thought patterns through guided exercises. This module has four sub-modules to be completed over a four-week period, including performing interactive programs on understanding automatic thought, cognitive distortions, and cognitive restructuring (B1), cognitive restricting practices for overgeneralization (B2), cognitive restricting practices for catastrophizing (B3), and additional repetitive practices (B4).
- Module C. Diaphragmatic Breathing: Body control training that induces relaxation by controlling breathing supported by instructional videos and practice logs. This module has four sub-modules to be completed over a four-week period, including learning diaphragmatic breathing and guided breathing training (C1), guided breathing training 1^st^ repetition (C2), guided breathing training 2^nd^ repetition (C3), and guided breathing training 3^rd^ repetition (C4).
- Module D. Progressive Muscle Relaxation (PMR): Body control training that sequentially tenses and relaxes the body muscles through instructional videos and practice logs. This module has two sub-modules to be completed over a two-week period, including learning and guiding muscle relaxation (D1) and guided whole body progressive relaxation (D2).
- Module E. Interoceptive Exposure: Guides patients to simulate feared bodily sensations to reduce avoidance and anxiety sensitivity. This module has two sub-modules to be completed over a two-week period, including understanding interoceptive exposure (E1) and specific exposure practices (E2).
- Module F. Mindfulness: Offers audio-guided meditation sessions with customizable backgrounds and voice options to foster present-moment awareness. This module has four sub-modules to be completed over a four-week period, including learning mindful breathing (F1), mindfulness practice 1^st^ repetition (F2), mindfulness practice 2^nd^ repetition (F3), and mindfulness practice 3^rd^ repetition (F4).
- Module G. Real-life Exposure: Supports stepwise real-life exposure to anxiety-inducing situations with structured planning and reflection. This module has three sub-modules to be completed over a three-week period, including identifying anxiety-provoking situations and imaginal exposure (G1), real-life exposure 1^st^ repetition (G2), and real-life exposure 2^nd^ repetition (G3).

The eight steps are structured as follows and are unlocked sequentially by a clinician's prescription.

- Step 1: Module A1, Module C1.
- Step 2: Module A2, Module B1, Module C2.
- Step 3: Module A3, Module B2, Module C3.
- Step 4: Module A4, Module B3, Module C4.
- Step 5: Module B4, Module D1, Module F1.
- Step 6: Module D2, Module F2, Module G1.
- Step 7: Module E1, Module F3, Module G2.
- Step 8: Module E2, Module F4, Module G3.

***Companion services***

These services are designed to help patients experiencing anticipatory anxiety or acute panic attacks as an easily accessible form of psychological first aid. The services provide a step-by-step practical guide based on the input of the user. It is designed to provide easy and immediate psychological support in time of crisis and to serve as an in-app safety net for patients in a state of panic. These services allow them to have a virtual companion that makes them feel as if someone is there with them and supporting them.

The services provide real-time coaching tailored to the user’s emotional state, such as anticipatory anxiety and panic attacks, referred to as “Preventing a Panic Attack” and “During a Panic Attack,” respectively. Patients are asked to complete an episode log, noting symptoms, medication use, and their feelings.

- The module of “Preventing a Panic Attack” provides patients with anticipatory anxiety with grounding exercises, cognitive prompts, and motivational voice guidance to reduce tension and prevent escalation.
- The module of “During a Panic Attack” helps patients with panic attacks initiate a structured coping sequence with paced breathing, attentional focusing, and step-by-step behavioral regulation.

***Care services***

These services enable patients to track lifestyle variables that affect panic symptoms:

- Medicine Record: Logging of prescribed medications with optional reminder alerts.
- Sleep Record: Daily records of sleep duration and subjective quality. Patients can see how long they slept and discuss it with a clinician.
- Exercise Record: Manual entry or automatic syncing with Google Health Connect to track activity type, duration, and intensity. As physical activity is vital for the treatment of anxiety disorder, this function was developed to motivate patients to work out more.
- Life-style Habits Record: Logging of consumption to identify behavioral patterns, such as caffeine intake, drinking, and smoking.

Each is recorded through daily inputs. These features are presented to patients in a format of monthly summaries to facilitate self-awareness and promote healthier routines. Additionally, self-rated daily mood score using a 5-point scale is provided to check improvements or changes in mood symptoms.

***Service assistance***

This section encompasses several supportive features designed to help patients navigate and engage with the app effectively. It includes:

- Reports and Feedback: This function provides weekly and monthly reports by combining data from all the modules. Patients are able to review their progress over time and reflect their pattern of the symptoms and behaviors. Training completion log, panic episode logs, lifestyle tracking trends, and mood fluctuations are presented. This promotes treatment adherence in addition to assisting patients in monitoring their progress. The report function is presented in a user-friendly format, a combination of simple graphs with a brief explanation. If clinicians provide written feedback, it is directly incorporated into the report interface within the app, promoting shared insight and reflection during follow-up sessions.
- More Information: Provides a concise overview of the app's purpose and structure, explaining how each feature contributes to treating panic disorder. This section also includes a first-time user guide on how to use the app.
- Frequently Asked Questions (FAQ): A list of questions and answers designed to address common user concerns. It is written in an informal tone to reduce user anxiety and improve app adherence. Patients can browse and search topics as needed, without relying on clinician contact for every query.

**2. The Management Console**

The management console is a web-based platform that allows clinicians to prescribe Waymed_panic services and monitor their adherence. This clinician-side console was designed to provide functions of patient registration, module assignment, and real-time monitoring to enhance continuity between in-person sessions and digital care. This console enables clinicians to review trends over time and offer timely feedback directly within the app. This feature helps facilitate collaborative treatment planning and allows psychiatric care to continue between appointments. As shown in the right side of Figure 1, it includes the following main elements.

- Managing Newly Registered Patients: Clinicians can create patient profiles, input demographic information, and issue app activation codes that link each patient to the supervising clinician.
- Prescribing Medication: The platform allows clinicians to specify prescribed psychotropic medications and intended usage patterns, which are reflected in the patient’s medication tracking section within the app.
- Prescription and Confirmation for Training: Clinicians can select CBT modules appropriate for the patient’s current clinical status, adjust the pace of delivery (e.g., session frequency and step progression), and provide comments. Session access is controlled through clinician-based unlocking.
- Checking: The dashboard displays a comprehensive calendar view showing individual progress across domains, including training session achievement rates, panic attack frequency and logs, daily symptom surveys, and weekly behavioral compliance metrics (e.g., lifestyle tracking).

**3. Additional Information**

The application was designed to be available on both Android and iOS operating systems and support Bluetooth connections with wearable devices (e.g., smartwatches).
