# Supplementary material for: Assessing the Usability of a Prescription-Based Mobile App for Patients With Panic Disorder and Its Management Console for Clinicians: Controlled User Study
Source: JMIR Form Res. 2025 Oct 17;9:e76843. doi: 10.2196/76843 (PMC12533929; doi:10.2196/76843)
Supplement: Multimedia Appendix 2 [file formative-v9-e76843-s002.docx]

| **[1] Running the app** | |  |  | 48. Go to the ‘Breathing Focus’ tab at the top of the ‘Results Report’.  49. Check your breathing focus score after training.  50. Explain verbally the meaning of the sentence below the graph: "60 to 100 means that you are focusing well on breathing."  51. Click the ‘Next and Confirm’ buttons to go to the ‘Comprehensive Evaluation’ page.  52. Click the ‘Next’ button on the ‘Comprehensive Evaluation’ page.  53. Click the ‘Confirm’ button, then click the ‘Select Other Training’ button. |
| --- | --- | --- | --- | --- |
|  | 1. Find the app icon and launch it.  2. Enter your email address and password.  3. Click the ‘Login’ button. |  |  |  |
| **[2] Training service – Studying about panic** | |  |  |  |
|  | 4. Go to the ‘Training Service’ on the home screen.  5. Go to ‘Studying about panic’.  6. Scroll down the screen to run the 5-session training.  7. Watch the training video you ran.  8. Solve Quiz 1.  9. Solve Quiz 2.  10. Select ‘Lecture Understanding’.  11. Click the ‘Finish Learning’ button.  12. Click the ‘Confirm’ button. |  |  |  |
|  |  |  | **[5] Training service – Progressive muscle relaxation** | |
|  |  |  |  | none |
| **[3] Training service – Correcting cognitive distortions** | |  | **[6] Training service – Interoceptive exposure** | |
|  | 13. Go to the ‘Training Service’ on the home screen.  14. Go to ‘Correcting Cognitive Distortions’.  15. Scroll down the screen and run the 2nd session: 'Negative Thought Finding Training'.  16. Watch the training video you ran.  17. Take the quiz on 'Antecedent Events', check your answers, and explain them verbally.  18. Take the quiz on 'Automatic Thoughts', check your answers, and explain them verbally.  19. Take the quiz on 'Results', check your answers, and explain them verbally.  20. When the evaluation record sheet appears, click ‘Confirm’.  21. Watch the training video you ran.  22. Proceed with 'Record Your Negative Thoughts: What am I afraid of?'.  23. When you're done writing, click ‘Next’ to move to the next page.  24. Proceed with 'Record Your Negative Thoughts: What would happen if that happened?'.  25. When you're done writing, click ‘Next’ to move to the next page.  26. Click the ‘Confirm’ button to complete the training. |  |  | 54. Go to the ‘Training Service’ on the home screen.  55. Go to ‘Body Control Training’.  56. Go to ‘Interoceptive Exposure’ tab.  57. Run the ‘Sensory Response Test’.  58. Check the precautions during training.  59. Click the ‘Run’ button on the training guidance screen.  60. Click the “Confirm’ button in the precautions during training.  61. Click the ‘Run’ button on the ‘Sensory Response Test’ screen.  62. Click the ‘Training Status’ at the top on the ‘Sensory Response Test List’ screen.  63. Check the graph at the top and the table at the bottom.  64. Describes the test type with the highest sensory intensity. |
|  |  |  | **[7] Training service – Mindfulness** | |
|  |  |  |  | 65. Go to the ‘Training Service’ on the home screen.  66. Go to ‘Mindfulness Training’.  67. Swipe the topic tab to the right to go to ‘Free Mindfulness Training’.  68. Run ‘Free Mindfulness: Find Inner Calm’.  69. Check the training precautions and click the ‘Confirm’ button.  70. Check the training settings arbitrarily and click the ‘Apply’ button.  71. Click the ‘Save’ button.  72. Touch the screen and click the ‘Training Settings’ icon on the upper right.  73. Change the background to the beach and click the ‘Apply’ button.  74. Touch the screen and click the ‘Training Instructions’ at the bottom.  75. Swipe the screen and check the training instructions until the end.  76. Click the ‘Confirm’ button to close the instructions.  77. Touch the screen and click the ‘Screen Transition’ icon on the upper right.  78. Touch the screen and click the ‘End Training’ button.  79. Click the ‘Go to Training Evaluation’ button.  80. Enter the tension level arbitrarily on the training evaluation screen.  81. Click the ‘Confirm’ button.  82. After checking the ‘Training Results Report’, verbally describe the subjective tension score after training.  83. Click the ‘Confirm’ button at the bottom of the result report.  84. Click the ‘Next’ button and the ‘Confirm’ button on the comprehensive evaluation page.  85. Click the ‘Select Other Training’ button. |
| **[4] Training service – Diaphragmatic breathing** | |  |  |  |
|  | 27. Go to the ‘Training Service’ on the home screen.  28. Go to ‘Body Control Training’.  29. Run ‘Daily Training’ in the ‘Diaphragmatic Breathing’ tab.  30. Watch the training video you ran.  31. Check the ‘Precautions during Training’.  32. Click the ‘Run’ button on the pre-evaluation screen.  33. Arbitrarily enter the current tension level and click the ‘Confirm’ button.  34. Click the ‘Run’ button on the training start guide page.  35. Arbitrarily set the training settings and click the ‘Confirm’ button.  36. Check the tutorial screen.  37. Click the ‘Play’ icon to start training.  38. Breathe according to the guide.  39. Click the ‘Confirm’ button on the practice completion page.  40. Click the ‘Run’ button for post-evaluation.  41. Arbitrarily enter the post-evaluation.  42. Click the ‘Run’ button on the ‘Results Report’ screen.  43. Look at the graph in the ‘Subject/Physical Tension’ tab and tell the change in score before and after training.  44. Go to the ‘Physical Tension’ tab.  45. Explain verbally the meaning of the sentence "60 to 89 times is normal tension" at the bottom of the page.  46. Go to the ‘Ease of Breathing’ tab at the top of the ‘Results Report’.  47. Explain verbally the meaning of the sentence above the graph: "This number describes how easy it is to breathe." |  |  |  |
|  |  |  | **[8] Training service – Real-life exposure** | |
|  |  |  |  | none |

| **[9] Companion service - Preventing a panic attack** | |  | **[12] Care service – Sleep record** | |
| --- | --- | --- | --- | --- |
|  | 86. Click the ‘Companion Service’ button at the top in the home screen.  87. Find the breathing guide on the ‘Preventing a Panic Attack’ page.  88. Run the breathing guide and set the training at will.  89. Click the ‘Apply’ button.  90. Tap the 'Play' button in the center of the screen to start the training.  91. Check the heart rate display at the top of the screen and explain it verbally.  92. Click the ‘Back’ button to go to the ‘Preventing a Panic Attack’ main page.  93. Find ‘Listen to the Words of Peace’ on the ‘Preventing a Panic Attack’ page.  94. Run ‘Listen to the Words of Peace’ and set it at will.  95. Click the ‘Apply’ button.  96. Run ‘Listen to the Words of Peace’ 3 times.  97. Click the ‘Back’ button to go to the ‘Preventing a Panic Attack’ main screen.  98. Click the ‘During a Panic Attack’ button at the bottom.  99. Click the ‘Next’ button on the ‘Companion Service’ screen.  100. Click the ‘Complete’ button on the ‘Take PRN Medicine’ screen. |  |  | 127. Go to the ‘Care service - Recording Sleep’ page.  128. Enter your sleep record arbitrarily.  129. Click the ‘Save’ button.  130. Return to the ‘Care service - Recording Sleep’ page.  131. Go to ‘Sleep Assistant’.  132. Click ‘Sleep Statistics’.  133. Go to sleep record for the month.  134. Verbally describe your actual sleep time for each week. |
|  |  |  | **[13] Care service – Exercise record** | |
|  |  |  |  | 135. Go to the ‘Care Service’ page.  136. Go to the ‘Recording exercise’.  137. Click the ‘Adding Exercise Record’ button.  138. Enter an exercise record arbitrarily.  139. Click the ‘Save’ button.  140. Click the ‘Start Exercise’ button on the ‘Recording exercise’ page.  141. Click the ‘Start Exercise’ button on the Guide page.  142. Click the 'End Exercise' button after 10 seconds.  143. Click the ‘End’ button on the pop-up asking whether to end the exercise.  144. Click the ‘Finish’ button on the ‘Exercise Summary’ page.  145. Click the ‘Confirm’ button on the ‘Exercise Complete’ pop-up. |
| **[10] Companion service - During a panic attack** | |  |  |  |
|  | 101. Click the ‘Chat with Helper’ button on the ‘During a Panic Attack’ page.  102. Click the ‘Talk with Helper’ button.  103. Type "I think I'm going to panic. What should I do?" and "Would it help to breathe slowly?" in the chat window.  104. Verbally explain the answer you get.  105. Click the ‘Back’ button to go to the ‘During a Panic Attack’ main page.  106. Click the ‘End During a Panic Attack’ button.  107. Click the ‘Record Now’ button on the ‘Mind Management after Ending Companionship’ screen.  108. Click the ‘Next’ button after arbitrarily recording the date and duration of the panic attack and taking PRN medication.  109. Select the ‘Feeling of Suffocation’ as the symptom you experienced and click the ‘Next’ button.  110. Select the degree of the ‘Feeling of Suffocation’ as mild and click the ‘Next’ button.  111. Select ‘Fatigue’ as the reason for worsening anxiety and click the ‘Next’ button.  112. Verbally explain the current value in the symptom severity graph on the ‘View Panic Record’ page.  113. Click the ‘Return to Preventing a Panic Attack’ button.  114. Click the ‘End’ button on the upper right of ‘Preventing a Panic Attack’ page.  115. Click the ‘Finish’ button.  116. Verbally explain the list of the functions for the activity on the companion service. |  | **[14] Care service – Life-style habits record** | |
|  |  |  |  | none |
|  |  |  | **[15]** **Service Assistance - Reports and feedback** | |
|  |  |  |  | 146. Go to ‘Report’ in the bottom menu bar of the home screen.  147. Click ‘STEP Basic’ on the top left to switch to the 'STEP-2' report.  148. Check the training progress (%) of the diaphragmatic breathing item in the training service and explain it verbally.  149. Click the ‘Back’ button to return to the home screen. |
|  |  |  | **[16] Service Assistance – More information** | |
|  |  |  |  | 150. Go to ‘More’ in the bottom menu bar.  151. Go to the ‘Alarm’ function.  152. Turn off ‘Alarm for Taking Medication’. |
|  |  |  | **[17] Service Assistance - Frequently Asked Questions** | |
|  |  |  |  | 153. Click the ‘More’ button and go to the ‘FAQ’ screen.  154. Tap the question "I forgot my authorization code."  155. Say what is displayed. |
|  |  |  |  | |
| **[11] Care service – Medicine record** | |  |  |  |
|  | 117. Go to the ‘Care service - Taking medicine’ page.  118. Select ‘Medication Record’.  119. Click the ‘Add’ button on the ‘PRN Medication Record’ screen.  120. Arbitrarily enter the medication name, dosage, time taken, and purpose of taking.  121. Click the ‘Save’ button.  122. Return to ‘Care service - Taking medicine’ page.  123. Go to ‘Medication Assistant’.  124. Go to ‘Medication Statistics’.  125. Click the ‘Month tab’.  126. Check and explain the number of days you took the medication differently from the prescription this month. |  |  |  |
