# Supplementary material for: Assessing the Usability of a Prescription-Based Mobile App for Patients With Panic Disorder and Its Management Console for Clinicians: Controlled User Study
Source: JMIR Form Res. 2025 Oct 17;9:e76843. doi: 10.2196/76843 (PMC12533929; doi:10.2196/76843)
Supplement: Multimedia Appendix 3 [file formative-v9-e76843-s003.docx]

| **[1] Running the console** | |  | **[5] Checking the comprehensive calendar** | |
| --- | --- | --- | --- | --- |
|  | 1. Enter your ID and password on the login screen.  2. Click the ‘Login’ button. |  |  | 21. Click the ‘>’ button on the patient list to move to the detailed view.  22. Find the ‘Training Date’ on the comprehensive calendar and say it. |
| **[2] Managing newly registered patients** | |  |  |  |
|  | 3. Click the ‘Add’ button on the patient list page.  4. Arbitrarily enter the patient’s name, assignment number, date of birth, contact information, gender, and clinical category.  5. Click the ‘Add’ button.  6. Check the new patient’s user code. |  | **[6] Checking the achievement rates** | |
|  |  |  |  | 23. Go to the ‘Care Service’ tab.  24. Find the drug information in the data details and achievement rate graph.  25. Touch the dot on the far right of the graph to indicate compliance.  26. Find the sleep information in the data details and achievement rate graph.  27. (Check average sleep efficiency) Explain the average sleep efficiency of the patient.  28. (Check the meaning of sleep efficiency) Click the sleep information icon on the right. |
| **[3] Prescribing medication** | |  |  |  |
|  | 7. Go to the ‘Medication Management’ tab.  8. Click the ‘Modify Prescription’ button.  9. Delete ‘Depas tablet 1 mg (Etizolam)’ from the PRN medicine.  10. Add ‘Xanax tablet 0.25 mg’ from the PRN medicine (for anxiety control, number: 1)  11. Click the ‘Save’ button. |  |  |  |
|  |  |  | **[7] Checking the panic attack-related data** | |
|  |  |  |  | 29. Go to the ‘Companion Service’ tab.  30. Explain the number of times indicated by the blue graph of the most recent week in the Companion Service details graph. |
| **[4] Prescription and confirmation for training** | |  |  |  |
|  | 12. Go to the ‘Training Service’ tab.  13. Click the ‘Prescribe’ button.  14. Designate ‘STEP 1 (Type A)’ to be used for 7 days starting today.  15. Click the ‘Add’ button.  16. Designate ‘STEP 2 (Type A)’ to be used for 7 days starting from the day after STEP 1 ends.  17. Enter the comment “Do breathing training 3 times a week” and click the ‘Add’ button.  18. Click the ‘Save’ button to prescribe the training.  19. Go to STEP 1 (1st) Prescription Details in the ‘Training Service’ tab.  20. Verbally explain the learning rate of the session that the patient did not complete. |  |  |  |
|  |  |  | **[8] Checking the daily survey results** | |
|  |  |  |  | 31. Find the daily basic survey graph of the survey details status.  32. Explain the depression and anxiety survey scores of the most recent week in the displayed graph. |
|  |  |  | **[9] Checking the weekly compliance results** | |
|  |  |  |  | 33. Go to the ‘Compliance Check’ tab.  34. Verbally explain the number of times Care Service and Companion Service were used in weeks 1 and 2. |
